# Isolation and characterization of a mycosubtilin homologue antagonizing *Verticillium dahliae* produced by *Bacillus subtilis* strain Z15

**DOI:** 10.1371/journal.pone.0269861

**Published:** 2022-06-13

**Authors:** Rongrong Lin, Qi Zhang, Li Yin, Yiwen Zhang, Qilin Yang, Kai Liu, Yingdian Wang, Shengcheng Han, Huixin Zhao, Heping Zhao

**Affiliations:** 1 Beijing Key Laboratory of Gene Resource and Molecular Development, College of Life Sciences, Beijing Normal University, Beijing, China; 2 Key Laboratory of Plant Stress Biology in Arid Land, College of Life Science, Xinjiang Normal University, Urumqi, China; College of Horticulture and Forestry (Dr YS Parmar University of Horticulture and Forestry), Nauni, Solan (HP), INDIA

## Abstract

*Bacillus subtilis* strain Z15 (BS-Z15) was isolated from the cotton field of Xinjiang, China, and characterized as an effective biocontrol agent antagonizing plant pathogen *Verticillium dahliae* 991 (VD-991). However, the chemical substance produced by BS-Z15 for resistance to VD-991 remains elusive. Here, a serial purification methods including HCl precipitation, organic solvent extraction, and separation by semi-preparative High-Performance Liquid Chromatography were performed to obtain a single compound about 3.5 mg/L from the fermentation broth of BS-Z15, which has an antifungal activity against VD-991. Moreover, Fourier Transform Infrared spectrum, Nuclear Magnetic Resonance Spectroscopy, and Tandem Mass Spectrometry analyses were carried out to finally confirm that the active compound from BS-Z15 is a mycosubtilin homologue with C17 fatty acid chain. Genomic sequence prediction and PCR verification further showed that the BS-Z15 genome contains the whole mycosubtilin operon comprising four ORFs: *fenF*, *mycA*, *mycB*, and *mycC*, and the expression levels of *mycA-N*, *mycB-Y* and *mycC-N* reached a peak at 32-h fermentation. Although mycosubtilin homologue at 1 μg/mL promoted the germination of cotton seed, that with high concentration at 10 μg/mL had no significant effect on seed germination, plant height and dry weight. Furthermore, mycosubtilin homologue sprayed at 10 μg/mL on two-week-old cotton leaves promotes the expression of pathogen-associated genes and gossypol accumulation, and greatly decreases VD-991 infection in cotton with disease index statistics. This study provides an efficient purification strategy for mycosubtilin homologue from BS-Z15, which can potentially be used as a biocontrol agent for controlling verticillium wilt in cotton.

## Introduction

*Verticillium dahliae* (VD) is a soil-borne plant fungal pathogen that causes verticillium wilt and infects over 600 plant species, including cotton (*Gossypium hirsutum)*, lettuce (*Lactuca sativa*), tomato (*Lycopersicum esculentum*) and strawberry (*Fragaria × ananassa Duch*.) etc, constituting severe threats to crops worldwide and yielding huge economic losses [1–5]. Researchers have developed resistant varieties, biocontrol agents, and chemical control methods against VD. However, the obscure infection mechanism in plant, lack of high resistant germplasm, the existence of long-term survival structure microsclerotia, and the broad ranges of hosts cause no effective control strategy established till now [4, 6–10]. In addition, the extensive application of chemical pesticides results in the increases of chemical-resistant pathogen, environmental pollution, and the disruption of agricultural ecosystem. Over the last couple of decades, biological control has attracted more attention, because of its advantages include low toxicity, low pollution, and efficiency, and numerous *Bacillus* species are widely used for biocontrolling a broad range of fungal plant pathogens [11–13]. Prior studies showed that one *Bacillus subtilis* strain Z15 (BS-Z15), isolated from cotton field of Xinjiang, China, had excellent antagonistic effect on VD-991, providing a positive role on controlling cotton verticillium wilt in China [14, 15]. However, the chemical substance produced by BS-Z15 for resistance to VD-991 is still unknown.

Previous studies showed that *Bacillus* species produces various lipopeptides for resistance to fungal pathogens, and according to their chemical structures, lipopeptides are mainly divided into three families: surfactins [16], fengycins [17], and iturins [18], each class exhibits different structural and functional characteristics [19]. Surfactins are composed of a cyclic heptapeptide connected to a C13-C16 β-hydroxy fatty acid chain, which act on biofilms, showing strong hemolytic activity, anti-bacteria and anti-viruse activities [20]. The fengycin class of lipopeptides are broad-spectrum antifungal agents and are particularly effective against plant pathogen [17, 21]. Fengycins are characterized by a cyclodecapeptide and a C15-C19 hydroxy fatty acid [22]. Deleu et al [23] showed that fengycins affect the structure and morphology of the membrane, further causing the death of target cells. The iturins share a common structure: a cyclic heptapeptide linked to a C14-C17 β-amino fatty acid chain [24]. Mycosubtilin belongs to the iturins family [25] and is considered as the most powerful antifungal compound [26–28]. Duitman *et al* [29] identified an operon consists of four ORFs, *fenF*, *mycA*, *mycB* and *mycC* from *Bacillus subtilis* strain ATCC6633, which encode enzymes for the biosynthesis of mycosubtilin. Moreover, using promoter replacement, promoter exchange and gene knock-out strategies, the genetic engineering strains for improving mycosubtilin production were constructed from ATCC6633, which was associated with the enhancement of the antifungal activities [26, 28].

In this study, a single active compound was isolated from the fermentation broth of BS-Z15 through the different purification methods with monitoring antifungal activity against VD-991. Furthermore, spectral analyses were performed to identify this chemical substance as a mycosubtilin homologue. In addition, genomic PCR and qRT-PCR was separately carried out to verify the existence of mycosubtilin operon and the expression of *mycA-N*, *mycB-Y* and *mycC-N* in BS-Z15 during the fermentation period, which is related to the production of mycosubtilin homologue. Furthermore, we found that spraying mycosubtilin homologue on the cotton leaves significantly reduced the disease index of cotton verticillium wilt. In addition, the expression of disease resistance genes and gossypol accumulation in the leaves were also promoted after spraying mycosubtilin homologue. Therefore, this study revealed that a mycosubtilin homologue produced by BS-Z15 has an antagonistic effect against VD-991, which contributed to controlling cotton verticillium wilt.

## Materials and methods

### Micro-organisms and culture conditions

BS-Z15 was previously isolated from cotton rhizosphere soil at Xinjiang province, China [14]. *Bacillus subtilis* strain 168 (BS-168) was kindly gifted by Kangcheng Pan at Sichuan Agricultural University [30]. The bacteria were cultured in beef extract–peptone medium (0.5% beef extract, 1% peptone, 0.5% NaCl, pH 7.2), and shaken at 220 rpm, 37°C for 12 h as the seed culture. Then, 2% seed culture was inoculated in 400 mL medium in 2-L flask, monitored with OD_595_ and disk antagonism test. For purification of the active compound, the bacteria were cultured in the 100-L fermentation tank contained 65 L beef extract–peptone medium at 37°C for 24 h, using peptone and beef extract as supplementary nitrogen source, 20% (v/v) HCl to adjust the pH, and the defoamer added to control tank pressure.

### Disk antagonism test against VD-991

The anti-VD-991 activity was detected by the agar-disk diffusion assay [15]. VD-991 were cultured in Czapek–Dox Medium at 25°C, 180 rpm for 24 h, and 20 μL of spore suspension of VD-991 (10^5 cfu/mL) were coated on Czapek–Dox Agar Medium. Then the circular filter paper (φ = 6 mm) soaked with 4 μL sample was placed on the plate. After incubated at 25°C for seven days, the inhibition zone diameter was measured. The experiments were repeated for at least three times and the average value of inhibition zone diameter was calculated.

### Isolation and purification of active compound from the fermentation broth of BS-Z15

The fermentation broth of BS-Z15 was collected by centrifugation to remove the bacterial residues, adjusted to pH 2.0 with 6 N HCl and refrigerated at 4°C overnight for precipitation. Then, the acid precipitates were collected by centrifugation with 4,000 g at 4°C and dissolved in 80% (v/v) pre-cooled acetone for 4 h at -20°C. The supernatant of acetone extraction was air-dried and dissolved in water. One volume of n-butanol was mixed thoroughly, the upper butanol phase was collected and evaporated as the crude compound. The crude compound was dissolved in DMSO and purified by a semi-preparative High-Performance Liquid Chromatography (semi-prep HPLC) system (C18, 5 μm, 250*10 mm, Hypersil GOLDTM, CA). Elution was performed with a gradient of 40%-50% acetonitrile (0.05% TFA, v/v) at a flow rate of 2 mL min^-1^ and monitored at 215 nm. Each peak was collected, concentrated and monitored by disk antagonism test. In addition, to quantify the production of active compound at different fermentation time, the peak area of HPLC spectrum was also monitored.

### The structure analysis of the active compound with Fourier Transform Infrared (FT-IR) spectrum, Nuclear Magnetic Resonance Spectrometry (NMR), and Tandem Mass Spectrometry (MS/MS)

The FT-IR spectral property of peak 4 (P4) fraction collected from semi-prep HPLC was recorded on the FT-IR spectrometer (NEXUS67, USA). The NMR (1H-NMR, 13C-NMR, 1H-1H COSY, HMBC, HSQC and ROESY) spectral analyses of P4 fraction were recorded on a Bruker AV500 spectrometer operating at 500 MHz, using DMSO-d6 (δH 2.51 ppm and δC 39.50 ppm) as a solvent. All NMR data were processed with MestReNova (version 6.2.0, Mesrelab, Spain). The MS/MS experiment was conducted on a Bruker MALDI-TOF 4700 mass spectrometer (Accelerating voltage: 20 KV, Matrix solution: 0.1% α-cyano-4-hydroxycinnamic acid in acetonitrile) [31].

### Validation of mycosubtilin biosynthesis genes in BS-Z15 genome

BS-Z15 genome was sequenced and deposited to NCBI database under the accession number QOCJ00000000, BioSample SAMN09582630 and BioProject PRJNA479656 [32]. Then, four ORFs for mycosubtilin synthase genes, *fenF*, *mycA*, *mycB*, and *mycC* from *Bacillus subtilis* ATCC6633 [29] were used to blast BS-Z15 genome to characterize the homologous genes. In addition, the encoded amino acids of these four ORFs from BS-Z15 genome were predicted by NRPS substrate predictor (NRPSsp) [33].

Bacterial genomic DNA was extracted by advanced CTAB method [34]. Three key genes, *mycB*, *ituA* and *sfp* separately associated with mycosubtilin, iturin A and surfactin biosynthesis, were validated by PCR amplification with BS-Z15 and BS-168 genome. PCR reactions were carried out on a MyCycler™ thermal cycler (Bio-Rad, USA) with a 15 μL reaction mixture. The amplification conditions were the initial denaturation at 95°C for 3 min, following with 35 cycles of 95°C for 15 s, 50°C for 30 s and 72°C for 25 s, and the final extension at 72°C for 5 min. Then, 10 μL of the amplification products was separated on a 1% agarose gel in 1×TAE buffer. The primers used are listed in S1 Table.

### Expression detection of mycosubtilin biosynthesis genes by quantitative real-time PCR (qRT–PCR)

Total RNA was isolated from BS-Z15 at different culture times using Total RNA Extraction Kit (Promega, USA) according to the manufacturer’s protocol. qRT-PCR was performed using an ABI Quant Studio 6 Flex Real-Time PCR System (Applied Biosystems, USA) with Power SYBR Green PCR Master Mix (Applied Biosystems). The thermal program was 10 min at 95°C, followed by 40 cycles of 15 s at 95°C and 60 s at 60°C. The relative expression of target gene was normalized to that of *16S rDNA* as the control. The relative expression at 0-h was set as 1.0 and value changes of more than twofold (> 2 or < 0.5) were indicated the significantly expression difference. Three biological replicates were performed per sample. The primers used are listed in S1 Table.

### Effect of mycosubtilin homologue on VD-991 growth in liquid culture

The spores of VD-991 (8*10^5 cfu/mL) were cultured with 0, 1, 5 and 10 μg/mL mycosubtilin homologue (filtrated sterilization with 0.22-μm filter membrane) in the 24-well culture plate containing 800 μL Czapek–Dox Medium, and shaken at 180 rpm at 25°C for 3 days. Then, the colony area of VD-991 was measured with ImageJ software (Version 1.53g4, National Institutes of Health, USA). The experiments were repeated at least three times and three replicates for each time.

### Effects of mycosubtilin homologue on cotton seed germination, seedling growth, and gene expression in leaves

The seeds of *Gossypium hirsutum* L *cv*. Xinluzao-72 (Xinluzao-92) were surface-sterilized with 2% sodium hypochlorite and cultured according to a prior study [35], separately soaked in 1, 10, and 100 μg/mL mycosubtilin homologue solution for 6 h, then germinated on gauze at 25°C for 52 h. Sterile water (ddH_2_O) was used as control. The germination experiments were repeated six times and 100 seeds for each time.

The cotton seeds were sown in plastic pots filled with sterile soil and placed in a greenhouse with day (25°C, 16 hr, light intensity at 6000 Lx)—night (21°C, 8 hr) rhythm for growth. 5 mL mycosubtilin homologue solution (10 μg/mL) was sprayed on two-week-old cotton leaves once a day for three consecutive days. ddH_2_O was used as the control. Then, plant height and dry weight of each plant were measured after 21 days.

In addition, two-week-old cotton leaves were treated with 5 mL mycosubtilin homologue solution (10 μg/mL) for 24 h, and collected for detecting the target gene expression. The expression levels of *Isochorismate synthase 1* (*ICS1*), *Pathogenesis-related protein-1* (*PR1*), *Phenylalnine-ammonia lyase* (*PAL*), and *2-Oxoglutarate/ Fe(II)-dependent dioxygenase* (*ODD*) were detected by qRT-PCR. *18S rDNA* was used as control. The gene expression level with the treatment of ddH_2_O was set as 1.0. Three biological replicates were performed. The primers used are listed in S1 Table.

### Gossypol content measurement in cotton leaves

The gossypol content was measured according to previous report with modification [36]. Two-week-old cotton leaves were sprayed with 5 mL mycosubtilin (10 μg/ml) once a day for three consecutive days, then dried for gossypol extraction. 50 mg dried leaves were ground in liquid nitrogen, extracted with 2.5 mL acetone by ultrasonification for 20 min, and centrifugation with 10,000 g at 4°C for 20 min. The supernatant was filtered with 0.22-μm filter membrane, injected to the HPLC system and eluted with 100% acetonitrile at 1 ml min^-1^, which was monitored on 234 nm. Different concentrations of gossypol were passed through HPLC and peak area was calculated for the standard curve. The experiments were repeated for at least three times.

### Effects of mycosubtilin homologue on cotton against VD-991 infection

To explore the biocontrol effects of mycosubtilin homologue on cotton verticillium wilt, pathogen incubated experiments were performed on the roots of two-week-old cotton seedlings [2, 37]. Briefly, the cotton seedlings were carefully taken out from the soil, the root were wounded with tip infection of VD-991 spore (4*10^5 cfu/mL) for 5 min, then replanted in the soil. After three days, 5 mL mycosubtilin solution (10 μg/mL) was sprayed on cotton leaves once a day for 3 days. The disease index (DI) was recorded every 7-days after VD-991 inoculation [38]. At least 20 cotton plants in each experiment, and three replicates are performed.

### Statistical analysis

Statistical analyses were performed using the GraphPad Prism 9.0 software. The statistical significances were determined by unpaired Student’s *t-test*. ns indicates no significant difference, *, ** and *** indicates statistical significance at P < 0.05, P < 0.01 and P < 0.001, respectively.

## Result

### Isolation and purification of a single antifungal compound produced by BS-Z15

**Fig 1:**
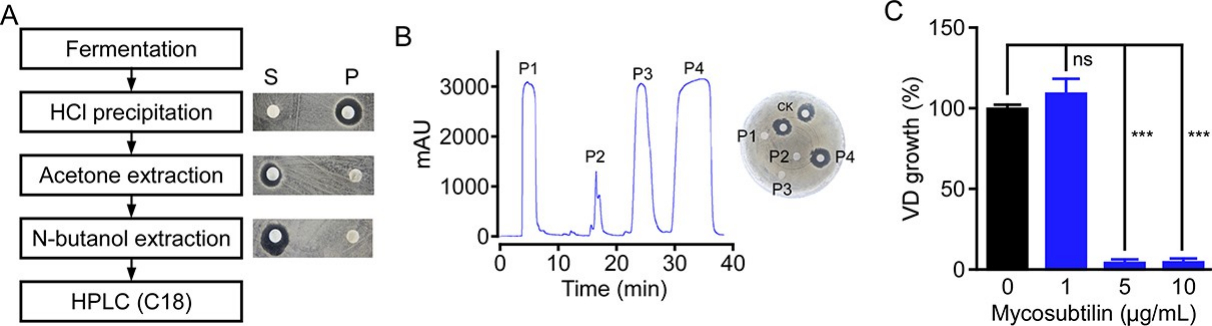
The isolation and purification of active compound resistance to *Verticillium dahliae* 991 (VD-991) from *Bacillus subtilis* Z15 (BS-Z15). (A) Flow chart showing the isolation and purification steps with BS-Z15 fermentation broth (left), and the active compound from each step were monitored by disk antagonism test (right). S indicates supernatant and P indicates pellet. (B) Semi-prep HPLC chromatogram of the crude compound collected from N-butanol extraction. The peaks P1 to P4 were collected for disk antagonism test against VD-991. CK is a positive control from fermentation broth. (C) The effect of P4 fraction on the growth of VD-991. The spores of VD-991 about 8*10^5 were cultured, and different concentrations of P4 fraction were added. The growth status was photographed after 3 days and the colony area was measured with ImageJ software. The experiments were repeated for three times. Data represents as mean ± SD (standard deviation). ns indicates no significant difference and *** indicates extreme significant at P < 0.001 (Student’s t-test).

To explore the mechanism on antagonizing VD-991 of BS-Z15, the isolation of the antifungal active substance produced by BS-Z15 becomes top priority. A modified purification strategy was performed with detecting anti-VD-991 activity. Firstly, the pellet from HCl precipitation of fermentation broth, the supernatant extracted by 80% (v/v) acetone (acetone phase) and the organic phase of n-butanol extraction showed strong anti-VD-991 activity (Fig 1A). These results indicated that the anti-fungal compound from BS-Z15 is an amphiphilic molecular. Then, the crude compound was separated by semi-prep HPLC. Four main peaks named P1 to P4, were eluted. Anti-fungal activity experiments showed that only P4 fraction exhibits a strong antagonistic activity against VD-991, which is yielded about 3.5 mg/L from the fermentation broth of BS-Z15 (Fig 1B). In addition, the active compound at 1 μg/mL has no effect on VD-991 growth, however, that at 5 and 10 μg/mL greatly inhibited VD-991 growth (Fig 1C). These results revealed that a single anti-VD-991 active compound is isolated from the fermentation broth of BS-Z15.

### Structure resolution of P4 fraction by FT-IR, NMR and MS/MS analysis

**Fig 2:**
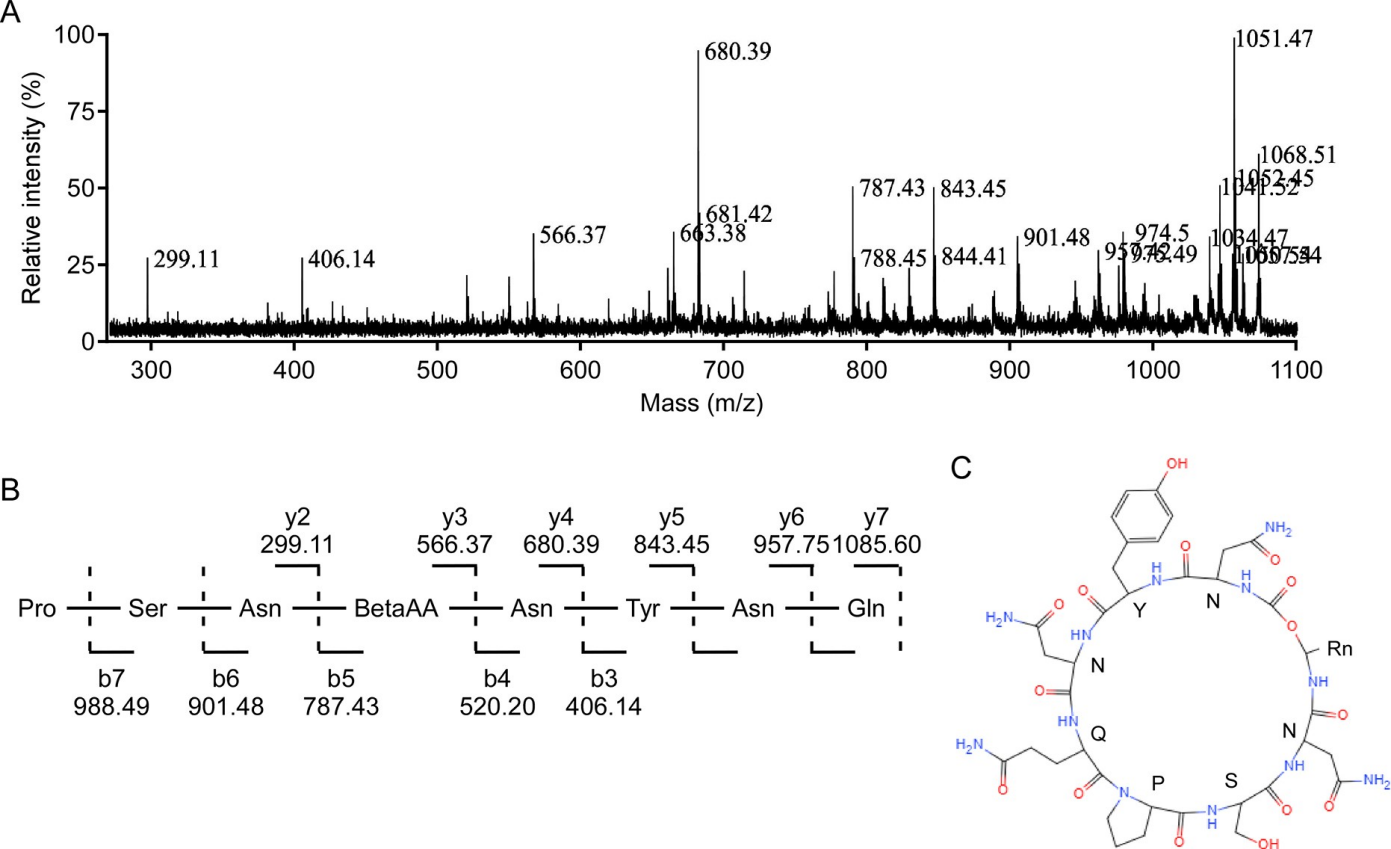
The structure resolution of mycosubtilin homologue isolated from BS-Z15. (A) The MALDI-TOF MS/MS fragmentations of P4 resulting from precursor ion of *m/z* 1085.60 [M+H]^+^. Data were analysed by Data Explorer software. (B) Schematic structure of mycosubtilin homologue with the b-type and y-type ionic fragments, corresponding to different positions of broken bonds from MS/MS spectrum shown in (A). (C) The chemical structure of mycosubtilin homologue. Seven amino acids were shown in the pattern and Rn represented a C14 alkyl chain.

In order to characterize the structure feature of that single active compound, FT-IR, NMR, and MS/MS spectra analyses was performed. Firstly, FT-IR spectrum showed a significant absorbance peak of amide bond N-H in 3320 cm^-1^, two C-H bands in 2925 cm^-1^ and 2854 cm^-1^, and hydroxyl groups of the amino acids C = O bands at 1663 cm^-1^, which suggest the existence of amide group in this compound (S1 Fig). Next, 1H-NMR, 13C-NMR, HMBC, HSQC, 1H-1H COSY, and ROESY spectra was adopted and summarized in Table 1. In the 1H-NMR (500 MHz, DMSO-d6) spectrum, 12 peaks of protons in NH/NH2 (δ 6.84–8.55) showed at low field, a benzene ring (δ 6.66, 7.02, d, J = 5.0 Hz, 2H) and 7 α-H from amino acids (δ 4.03–4.61) was also identified (S2 Fig). In the 13C-NMR (150 MHz, DMSO-d6) spectrum, 12 peaks of carbonyl from C = O (δ 170.4–174.7), a benzene ring (δ 128.3, 130.3, 130.3, 115.5, 115.5, 156.3), 7 α-C from amino acids (δ 50.5–60.5), 2 methyl groups (δ 23.0, 19.6) was also identified (S3 Fig). Combined analysis of 1H-NMR and 13C-NMR showed multiple methylene structures (δ 1.06–1.51) in high field, suggesting that there may be a long aliphatic chain with two methyl groups at the end (δ 0.83, dd, 6H). Combined analysis of 2D spectrum 1H-1H COSY, HMBC, HSQC and ROESY (S3–S7 Figs), it can be confirmed that this compound contains 3 asparagine (Asn), 1 tyrosine (Tyr), 1 Aminoamide (Gln), 1 proline (Pro) and 1 serine (Ser) (Table 1). Finally, the MS/MS spectrum was performed with the precursor ion at *m/z* 1085 [M+H]^+^. After analysing b type and y type ions, the amino acid sequence of Pro-Ser-Asn was obtained from b5, b6, b7 and y2, the sequence of Asn-Tyr-Asn-Gln was obtained from y3, y4, y5, y6, y7 and b3. Fragments of open peptide showed that the amino acid sequence could be Pro-Ser-Asn-fatty acid-Asn-Tyr-Asn-Gln, which is corresponding well to C17 mycosubtilin, respectively (MW: 1084.596 Da, C_51_H_80_O_14_N_12_) (Fig 2). Therefore, this study first characterized an anti-VD991 mycosubtilin homologue from the fermentation broth of BS-Z15.

**Table 1:** Nuclear Magnetic Resonance (NMR) spectral property of P4 fraction isolated by semi-prep HPLC with Bruker AV500 spectrometer.

Moiety	Position	δ_C_	δ_H_	^1^H-^1^H COSY	Selected HMBC	Selected ROESY
Asn1	1	50.5	4.42	2	2,3,4	
	2	31.2	2.14		1,3,4	
	3	174.7			1,2	
	4	173.6			2	
	1-NH		8	1	1	1,36,37,δH 8.55
	3-NH2		7.24		3	2
Tyr2	5	56.9	4.03	6		6,8,12
	6	35.6	2.92/2.73		5,7,8,12,13	
	7	128.3				
	8, 12	130.3	7.02			5,6,9,11
	9, 11	115.5	6.66			8,12,δH 9.22
	10	156.3				
	13	171.7				
	5-NH		8.55	5	4,5	
	10-OH		9.22			
Asn3	14	51.2	4.42	15	17	
	15	36.8	2.3/2.2		14,16,17	
	16	171.7				
	17	171.7				
	14-NH		8.03	14	13	5,14,δH 7.14,δH 8.55
	16-NH2		6.92/6.91		15,16	
Gln4	18	50.6	4.42	19	19,20	19,20
	19	36.6	2.5		20,21	
	20	36.5	2.3		21	
	21	171.7				
	22					
	18-NH		7.14	18		14
	21-NH2		6.84/6.9			19
Pro5	23	60.5	4.29	24,26	24,25,27	24
	24	25.2	2.14		26	
	25	29.4	1.97/1.85			
	26	26.8	1.77/1.87		27	23
	27	173				
Ser6	28	55	4.21	29	29,30,27,δH 4.81	
	29	60.9	3.65/3.57		30,δH 4.81	
	30	170.4				
	28-NH		8.49	28	27,28	23,28
	29-OH		4.82			
Asn7	31	50.5	4.61	32	32,33,34	32
	32	37.7	2.63/2.24		33,34	
	33	171.7				
	34	171.1				
	31-NH		7.84	31	30	28,31
	33-NH2		7.27		33	31
β-AA	35	46.8	3.95		36	36
	36	41.9	2.33/2.27		37	
	37	171.7				
	35-NH		7.11			
Fatty chain	38	34.9	1.35/1.37			
	39–47	25–29.9	1.06–1.99		35	
	48	27.8	1.47		36	36
	49	34.2	1.26			
	50,51	11.7/19.6	0.83			36,37

### Gene mining and PCR-validation of mycosubtilin biosynthesis genes in BS-Z15

**Fig 3:**
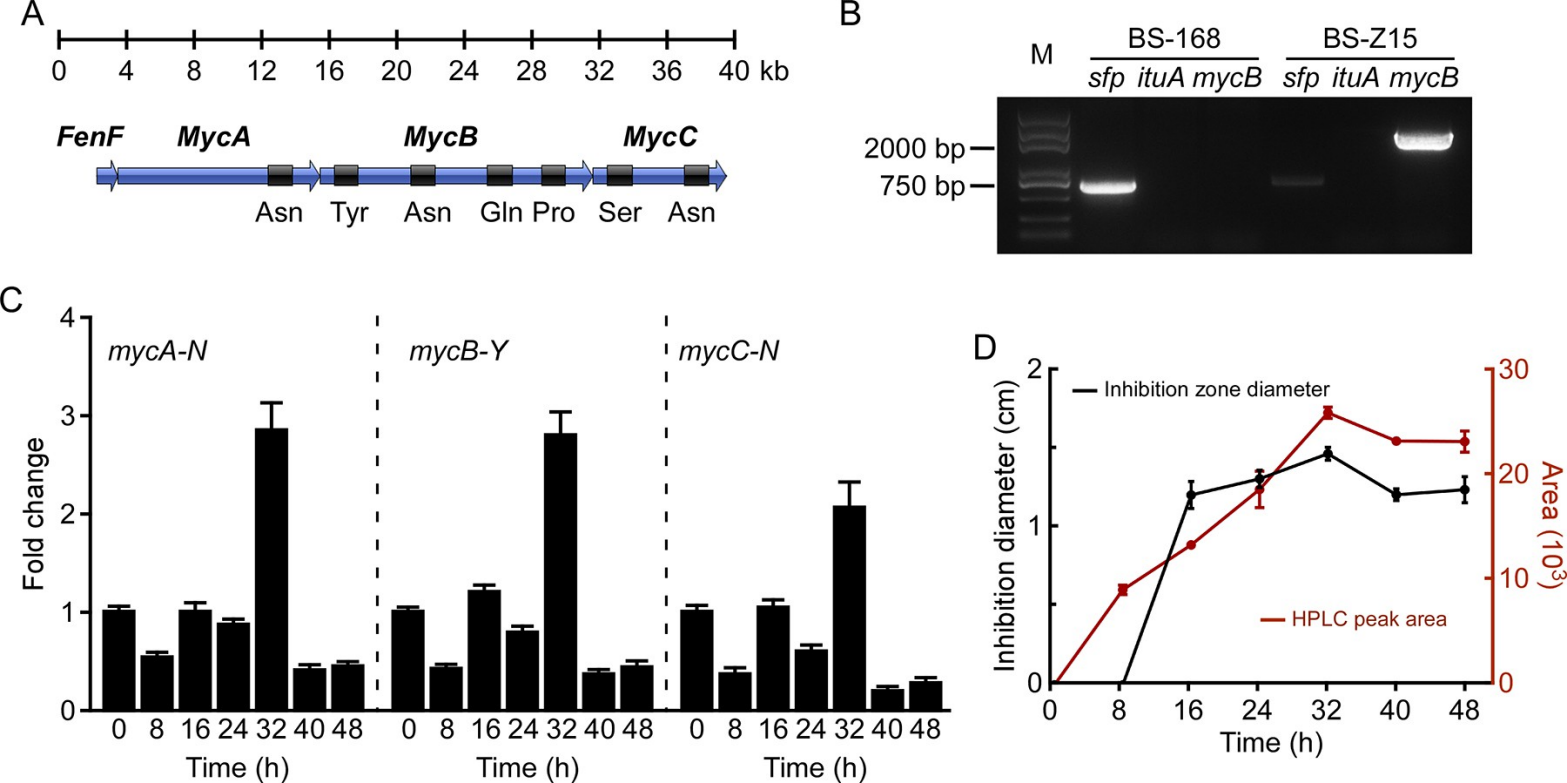
Gene mining and expression identification of mycosubtilin biosynthesis genes in BS-Z15 genome. (A) The gene mining for mycosubtilin biosynthesis from BS-Z15 genome. Diagrammatic representation of the entire mycosubtilin operon comprising four ORFs, *fenF*, *mycA*, *mycB*, and *mycC*. The amino acids specified by the operon are indicated. (B) DNA fragments of *sfp*, *ituA* and *mycB* related to the productions of surfactin, iturin A and mycosubtilin were detected by genomic PCR in BS-Z15 and BS-168 strains, respectively. M is a DNA marker. (C) The transcriptional levels of *mycA-N*, *mycB-Y* and *mycC-N* in BS-Z15 monitored by qRT–PCR at different culture times. The relative expression of target gene was normalised to that of *16S rDNA*, and the expression value at 0 h was set as 1.0. Values are means ± SD of three independent experiments. (D) The mycosubtilin content and anti-VD-991 activity in fermentation broth of BS-Z15 were monitored during the fermentation period. Mycosubtilin content was defined by HPLC peak area (red line), and anti-VD-991 activity was characterized by disk antagonism test (black line). Data represent mean ± SD (n = 3).

After blasting BS-Z15 genome, four ORFs for mycosubtilin synthase genes, *fenF*, *mycA*, *mycB*, and *mycC*, spanned about 38 kilobases were annotated in BS-Z15 genome (Fig 3A) which is similar to previous study [29]. With further explored these four ORFs by NRPSsp, one amino acid Asn was predicted as the substrate of MYCA, four amino acids Tyr, Asn, Gln, and Pro as the substrates of MYCB, and two amino acids Ser and Asn as the substrates of MYCC, respectively, which is exactly same as the components of mycosubtilin homologue (Fig 3A and S2 Table).

A prior study showed that BS-168 produces surfactin, but not mycosubtilin [39]. Therefore, the genome of BS-168 was used as a control to characterize *mycB* and *sfp*. As shown in Fig 3B and S1 File, *mycB* was amplified in BS-Z15 genome, but not in BS-168. In addition, a strong band of *sfp* fragment was amplified in BS-168 genome, but only a weak band in BS-Z15, and *ituA* fragment can’t be amplified in both genome of BS-Z15 and BS-168. These results indicate that BS-Z15 mainly produces a mycosubtilin homologue, but not iturin A and surfactin. Moreover, we found that the expression of *mycA-N*, *mycB-Y* and *mycC-N* reached the maximum at 32 h and then decreased during fermentation period (Fig 3C), which is agreement with the mycosubtilin homologue production and its anti-VD-991 activity during culture time (Fig 3D).

### The effects of mycosubtilin homologue on plant growth, gene expression, gossypol accumulation and resistance to verticillium wilt in cotton

**Fig 4:**
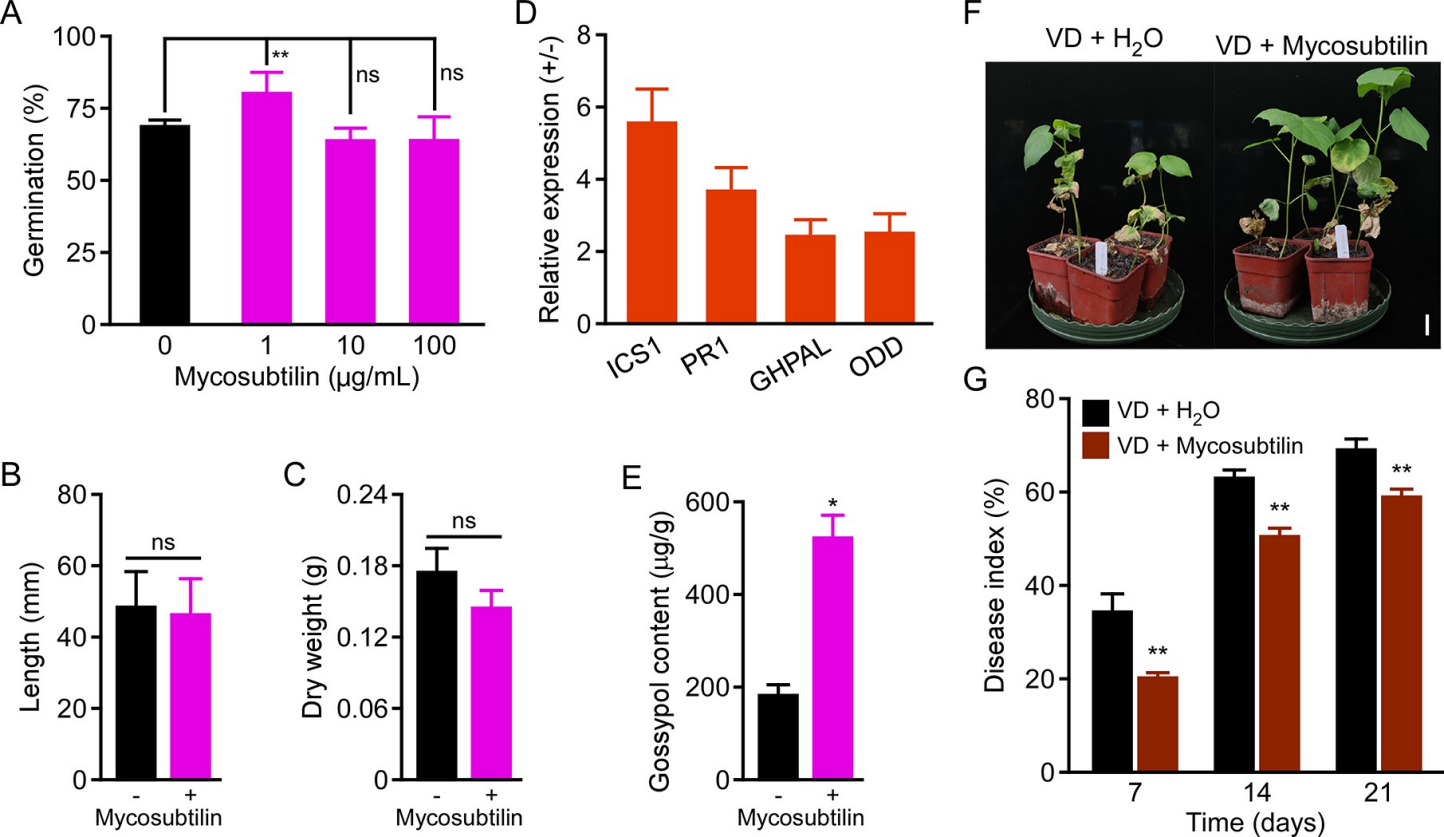
The effect of mycosubtilin homologue on plant growth, the expression of pathogen-associated genes, and resistance to verticillium wilt in cotton seedlings. (A) The germination of cotton seeds after treated with 0, 1, 10, and 100 μg/mL mycosubtilin homologue solution. The experiments were conducted for at least 100 grains in each group and with 6 replicates. Data were shown as mean ± SD. (B,C) Plant height (B) and dry weight (C) of cotton seedlings were measured after sprayed 5 mL mycosubtilin homologue (10 μg/mL) on two-week-old cotton leaves for 21 days. The data represent the mean ± SD (n = 3). (D) The transcriptional levels of *ICS1*, *PR1*, *PAL* and *ODD* in cotton leaves with mycosubtilin homologue treatment for 24 h were monitored by qRT-PCR. The expression level of target gene was normalized to that of *18S rDNA* as the control. The relative expression for each gene with the treatment of ddH_2_O was set as 1.0. Values are mean ± SD (n = 3). (E) Gossypol contents in cotton leaves with 5 mL mycosubtilin homologue (10 μg/mL) treatment. Data were shown as mean ± SD (n = 3). (F) and (G) The biocontrol effects of mycosubtilin homologue on cotton verticillium wilt. Photograph (F) was taken at 21 days post inoculation (dpi). Disease indexes of the cotton seedlings (G) were determined at 7, 14 and 21 dpi. The data represent the mean ± SD (n = 3). In this figure, ns indicates no significant difference and ** indicates extreme significant at P < 0.01 (Student’s t-test).

Firstly, the effect of mycosubtilin homologue on cotton seed germination and seedling growth was monitored. With the treatment of 1 μg/mL mycosubtilin homologue, the germination ratio was higher than control, but high concentration at 10 and 100 μg/mL mycosubtilin homologue had the similar germination percentage as control, respectively (Fig 4A). Furthermore, the plant height and dry weight of cotton seedlings showed no significant difference between the treated of 10 μg/mL mycosubtilin homologue and control (Fig 4B and 4C). These results revealed that low concentration of mycosubtilin homologue promote the seed germination, but high concentration has no obvious effect on cotton seed germination and seedling growth.

Next, we detected whether mycosubtilin homologue regulates the expression of pathogen-associated genes and gossypol accumulation in cotton leaves. qRT-PCR showed that the expression of *ICS1*, *PR1*, *PAL* and *ODD* are greatly upregulated in cotton leaves in response to mycosubtilin homologue treatment (Fig 4D). Moreover, gossypol content was also higher after treated with mycosubtilin homologue than control (Fig 4E). Finally, we found that the disease index of cotton verticillium wilt was greatly decreased after spraying mycosubtilin homologue on cotton leaves (Fig 4F and 4G). These results indicated that mycosubtilin homologue produced by BS-Z15 control cotton verticillium wilt by enhancing the expression of pathogen-associated genes and the gossypol accumulation, providing a potential effective biocontrol agent for cotton verticillium wilt.

## Discussion

Mycosubtilin is one member of the iturin family, which is composed of a cyclic heptapeptide linked to a C14-C17 β-amino fatty acid chain [24, 25], and widely proved as a broad antifungal spectrum, including *Saccharomyces carlsbergensis*, *Aspergillus niger* and *Fusarium moniiforme*, etc [26–28, 40]. However, fewer research on mycosubtilin controlling plant fungal diseases was mainly due to its purification difficulty, low production and seldom produces by strains [41]. In this study, a mycosubtilin homologue was purified from the fermentation broth of BS-Z15 and its anti-VD-991 activity was also confirmed. Moreover, the expression of pathogen-associated genes and gossypol accumulation were also promoted and the disease index of cotton infected by VD-991 was decreased after spraying by mycosubtilin homologue. These results suggested that mycosubtilin homologue from BS-Z15 not only directly inhibits the growth of VD-991, but also promotes the expression of pathogen-associated genes and gossypol accumulation for resistance to VD-991, which providing a potential agent for controlling verticillium wilt of cotton.

Previous studies showed that both BS-Z15 and its fermentation broth possess the strong anti-VD-991 activity [14, 15], indicating that the active compound from BS-Z15 is water-soluble and extracellular-secreted, indicating a basic purification strategy. Therefore, HCl precipitation was first performed to concentrate the compound. In order to remove the disturbances of pigments and nuclear acids, 80% (v/v) acetone was used to extract the pellet. Next, we tried different organic solutions, e.g. n-butanol and methanol to dissolve the active compound, and found that n-butanol is a better solvent for this compound than methanol. Because of this compound is an amphiphilic molecule, silica gel chromatography with different mobile phases and ion exchange chromatography were performed to isolate the active compound, however, the effect of purification is very poorly. Finally, HPLC equipped with C18 column was used to purify this compound. More importantly, the disk antagonism test against VD-991 was performed to track the active compound for each step. Because the structure and chemical character of mycosubtilin were high similar in iturin group, this optimized and convenient methods can be used for the purification of other members of iturin produced by Bacillus species.

In this study, an operon consisting of four ORFs, *fenF*, *mycA*, *mycB* and *mycC* was firstly characterized by annotated the genome sequence of BS-Z15, which is highly agreement with that of ATCC6633 [29]. Next, the substrates of these enzymes encoded by four ORFs were predicted using NRPSsp program [33], and we found that the substrate amino acids were consistent with the cyclic peptide sequences of mycosubtilin homologue that was characterized by the structural analysis with spectroscopy methods. These result proved the previous report of mycosubtilin biosynthesis carried out by non-ribosomal peptide synthetases (NRPSs) [42]. In addition, a previous study showed that BS-168 produces two lipopeptides, surfactin and fengycin, but not mycosubtilin [39]. Genomic PCR results further revealed that BS-Z15 possess the *myc* gene, but no *sfp* gene, indicating that BS-Z15 produces mycosubtilin, but not surfactin. Using genetic manipulation strategies, the different genetic engineering strains were constructed from ATCC6633 to improve the productions of surfactin and different mycosubtilin isoforms [26–28]. Here, we characterized a single mycosubtilin homologue from BS-Z15, which conferred resistance to VD-991. Therefore, to improve the production of mycosubtilin homologue in BS-Z15 or characterize the anti-VD-991 activity of ATCC6633 will be the priority works in the future.

Mycosubtilin has been identified as an antifungal lipopeptide, which directly interacted with and induced the striking changes in the organization and morphology of membrane lipid layers [43, 44]. Here, we showed that mycosubtilin effectively inhibit the growth of VD-991 in liquid culture or after spraying on cotton leaves, which suggested that it can be used as a potential biocontrol agent for controlling cotton verticillium wilt diseases. In addition, we found that mycosubtilin homologue promoted the expression of pathogen-associated genes, such as *ICS1*, *PR1*, *PAL* and *ODD*, and gossypol accumulation in cotton leaves. Similar to that, Farace et al [11] found that mycosubtilin stimulated grapevine innate immune responses, including salicylic acid (SA) and jasmonic acid (JA) signalling pathways mediated gene expression. These results provide two potential synergistic mechanisms on cotton verticillium wilt diseases: After sprayed on leaves, mycosubtilin homologue can be transported to vascular tissue to directly against *Verticillium dahliae* and activated the innate immune responses for pathogen resistance, which needs a deep further study to characterize.

## Supporting information

S1 Fig Fourier Transform Infrared (FT-IR) spectral property of P4 fraction isolated by semi-prep HPLC with Fourier transform infrared spectrometer. (PDF)

S2 Fig The 1H-NMR spectra of active substance isolated from BS-Z15. (PDF)

S3 Fig The 13C-NMR spectra of active substance isolated from BS-Z15. (PDF)

S4 Fig The HSQC spectra of active substance isolated from BS-Z15. (PDF)

S5 Fig The HMBC spectra of active substance isolated from BS-Z15. (PDF)

S6 Fig The COSY spectra of active substance isolated from BS-Z15. (PDF)

S7 Fig The ROESY spectra of active substance isolated from BS-Z15. (PDF)

S1 Table Primers used for genomic PCR and qRT-PCR. (PDF)

S2 Table Predicated amino acid encoded by mycosubtilin synthase genes in BS-Z15 genome based on NRPSsp. The four ORFs of Mycosubtilin synthase genes from BS-Z15 were translated to FenF, MycA, MycB and MycC proteins, then analysed by NRPSsp based on the adenylation domain. The start position, end position and predicted substrate were showed in the table. FenF protein has no any predicted adenylation domain.
(PDF)

S1 File Raw gel image. (PDF)
