# Supplementary figures and images for: Isolation and characterization of a mycosubtilin homologue antagonizing Verticillium dahliae produced by Bacillus subtilis strain Z15
Source: PLoS One. 2022 Jun 13;17(6):e0269861. doi: 10.1371/journal.pone.0269861 (PMC9191732; doi:10.1371/journal.pone.0269861)

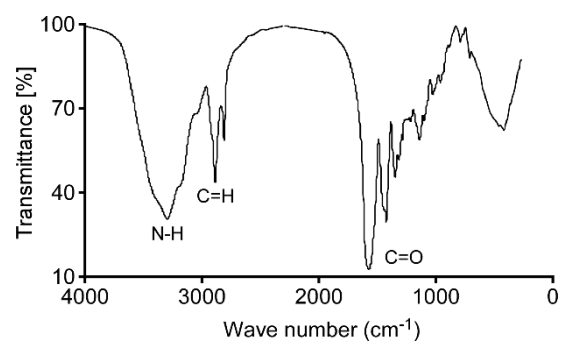

Supplement: S1 Fig — (PDF) [file pone.0269861.s001.pdf]

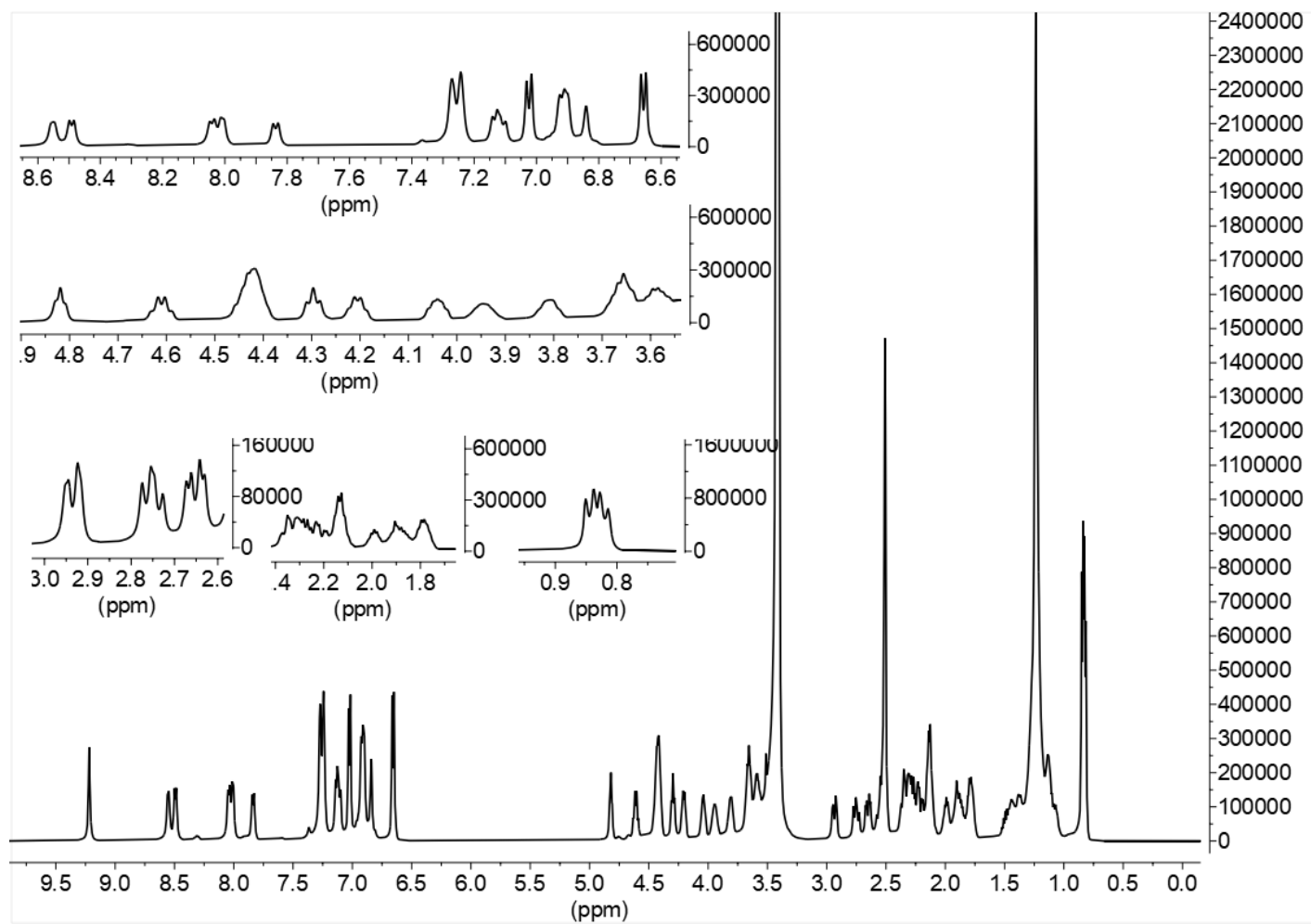

Supplement: S2 Fig — (PDF) [file pone.0269861.s002.pdf]

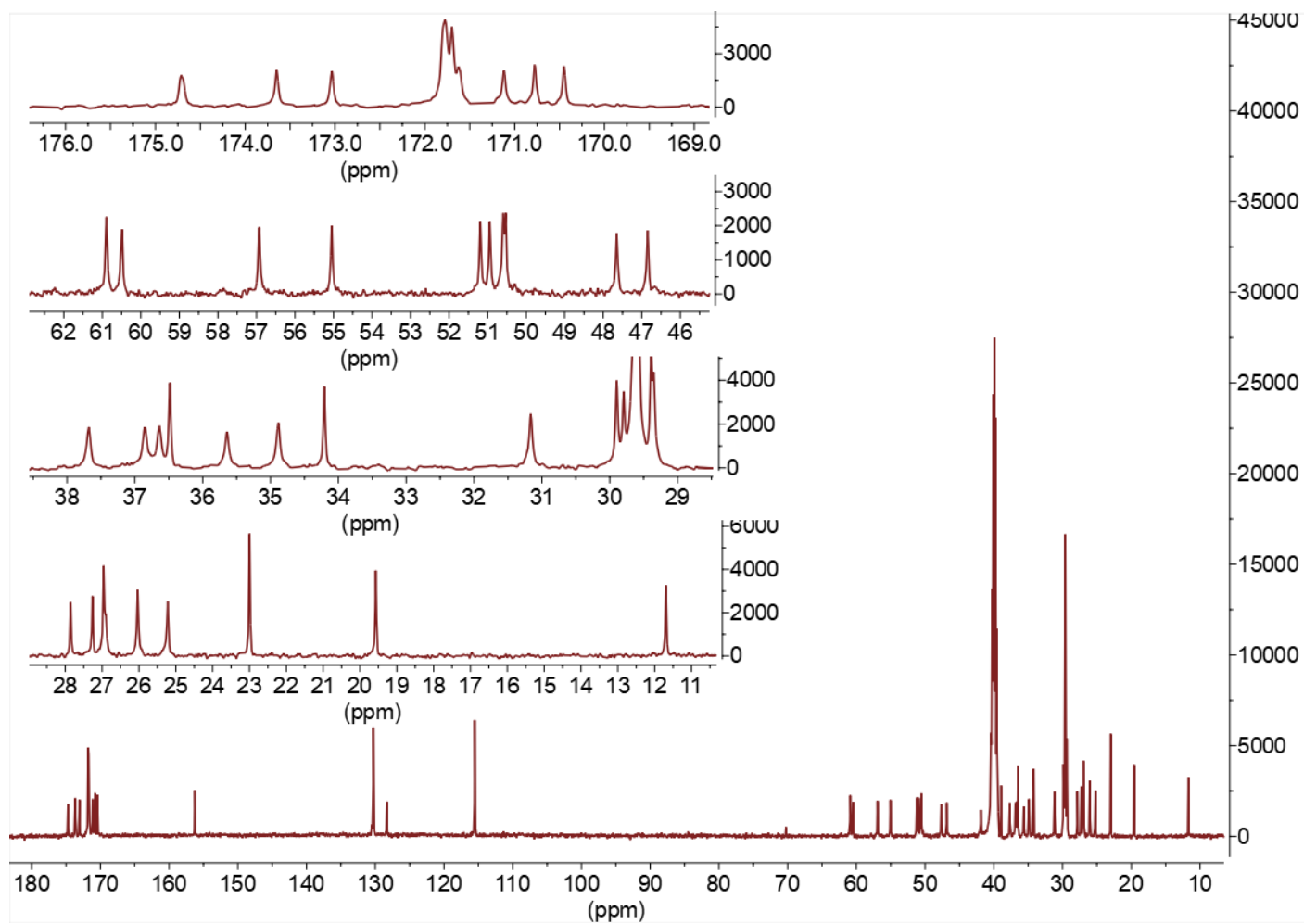

Supplement: S3 Fig — (PDF) [file pone.0269861.s003.pdf]

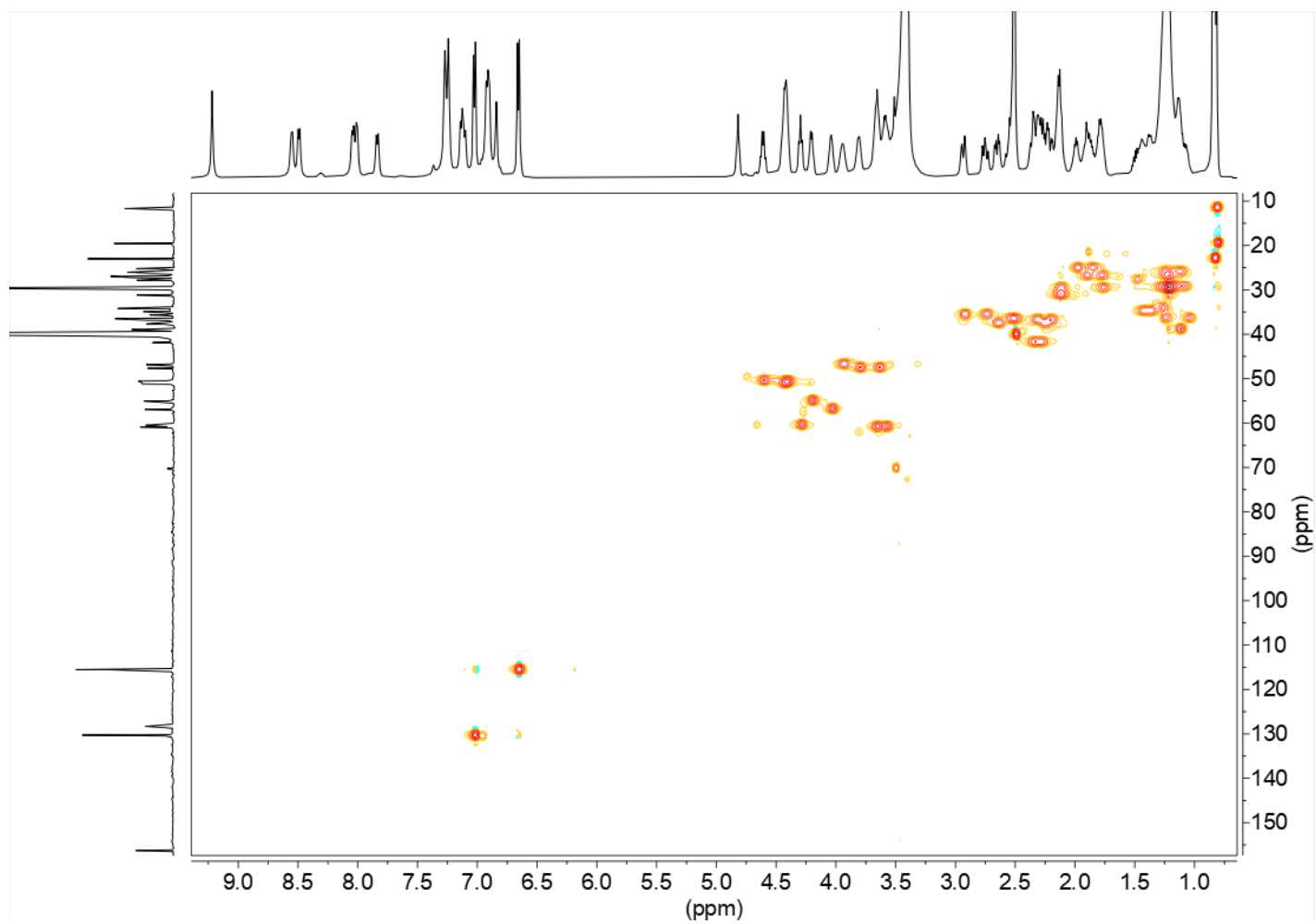

Supplement: S4 Fig — (PDF) [file pone.0269861.s004.pdf]

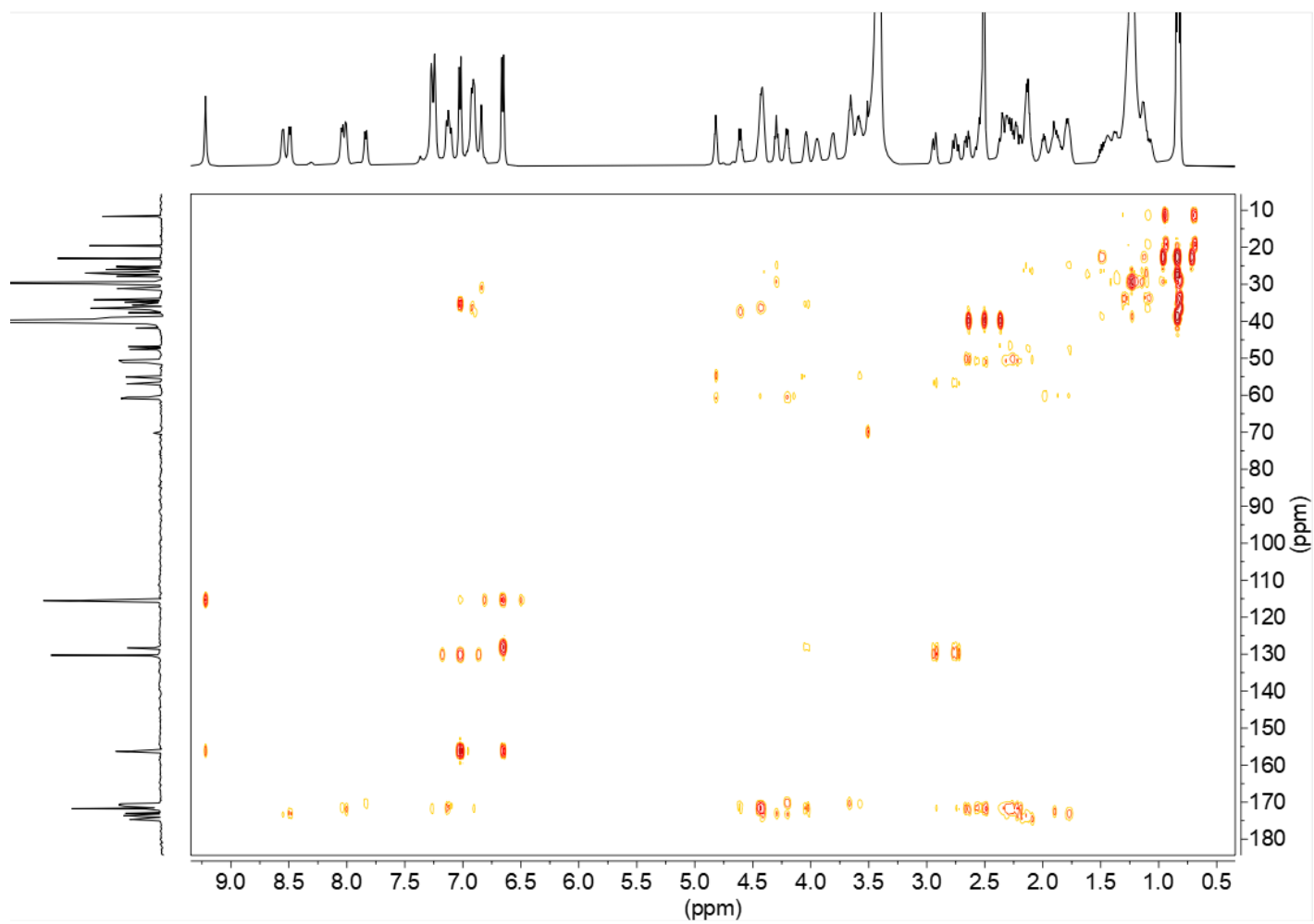

Supplement: S5 Fig — (PDF) [file pone.0269861.s005.pdf]

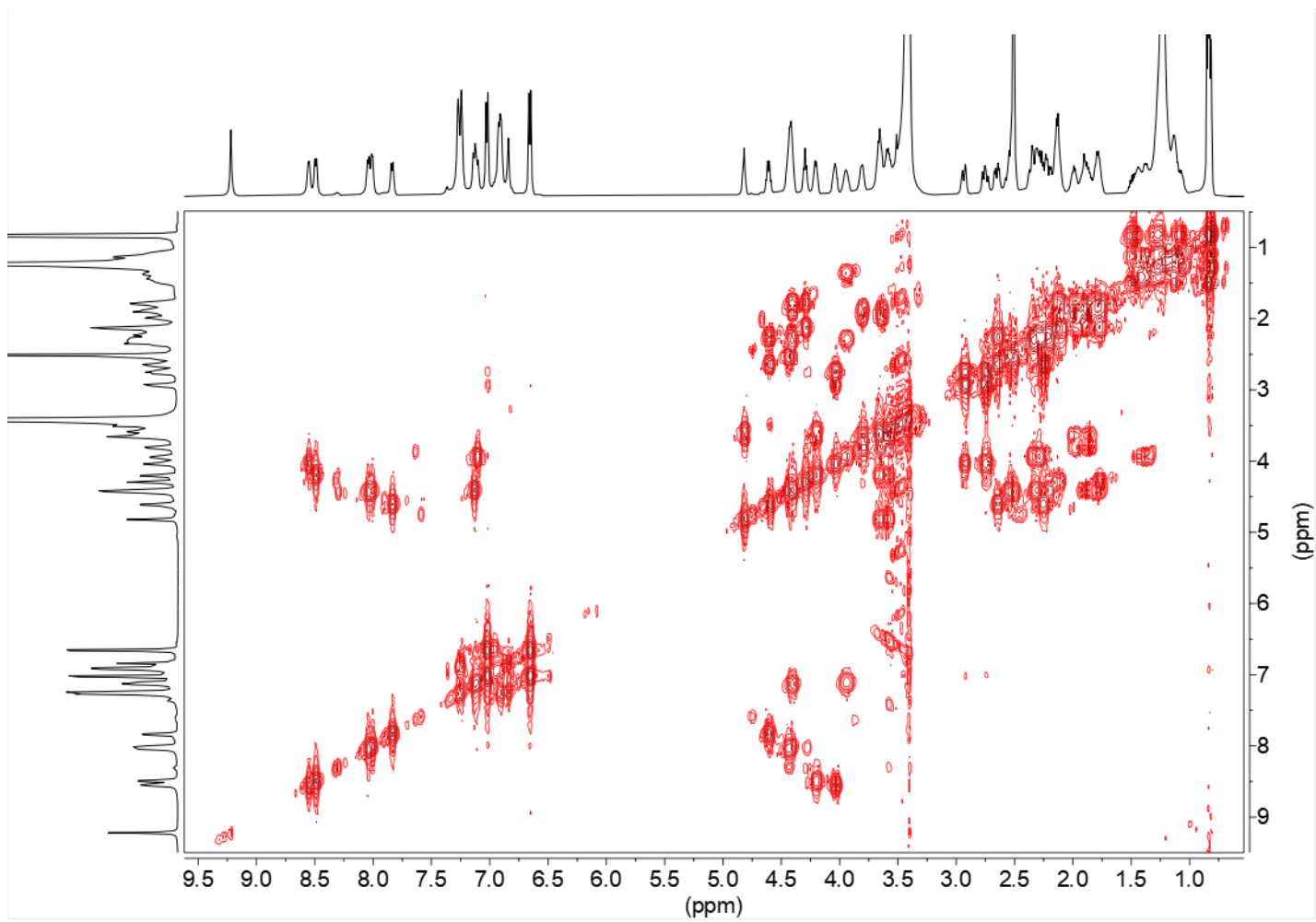

Supplement: S6 Fig — (PDF) [file pone.0269861.s006.pdf]

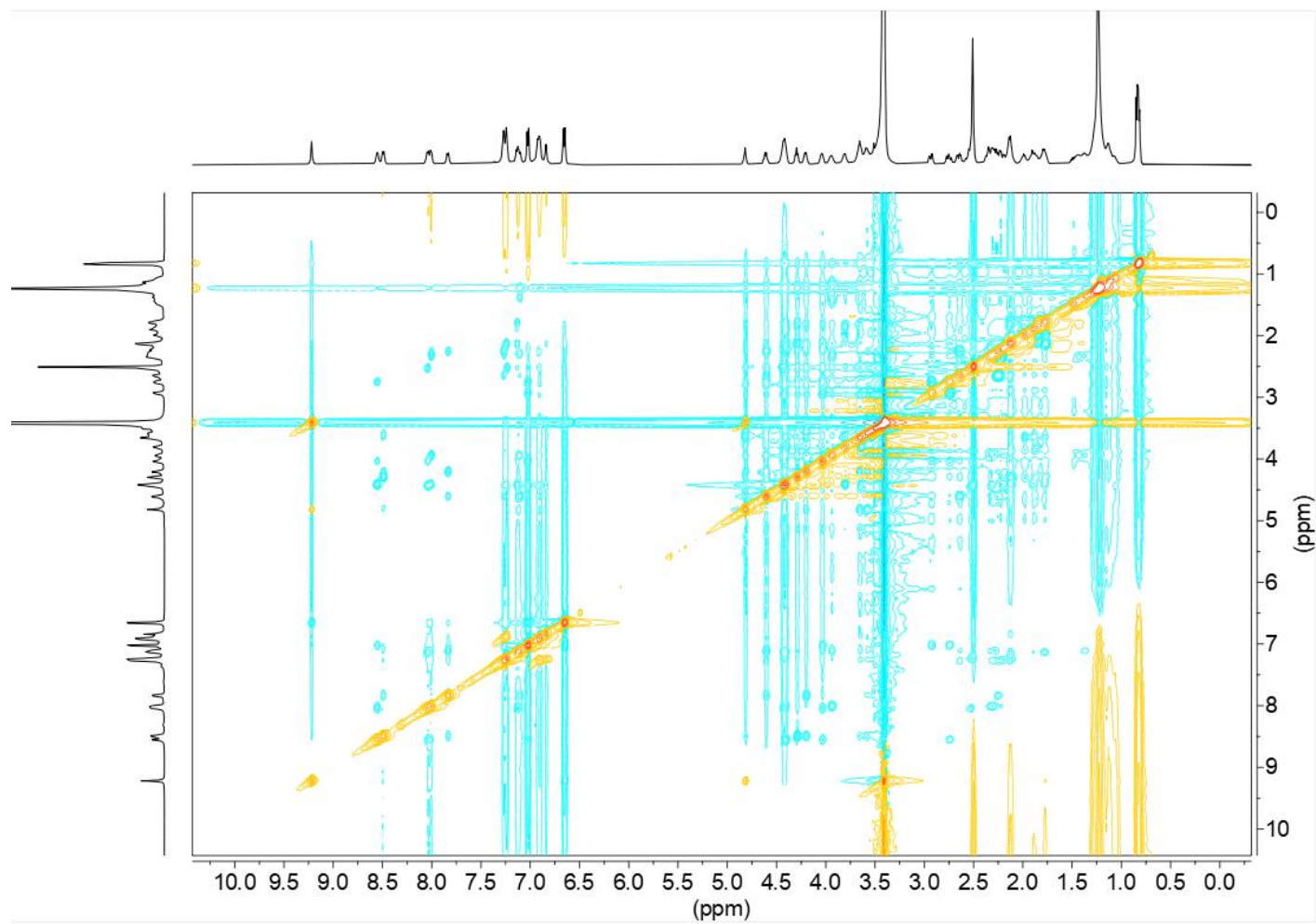

Supplement: S7 Fig — (PDF) [file pone.0269861.s007.pdf]

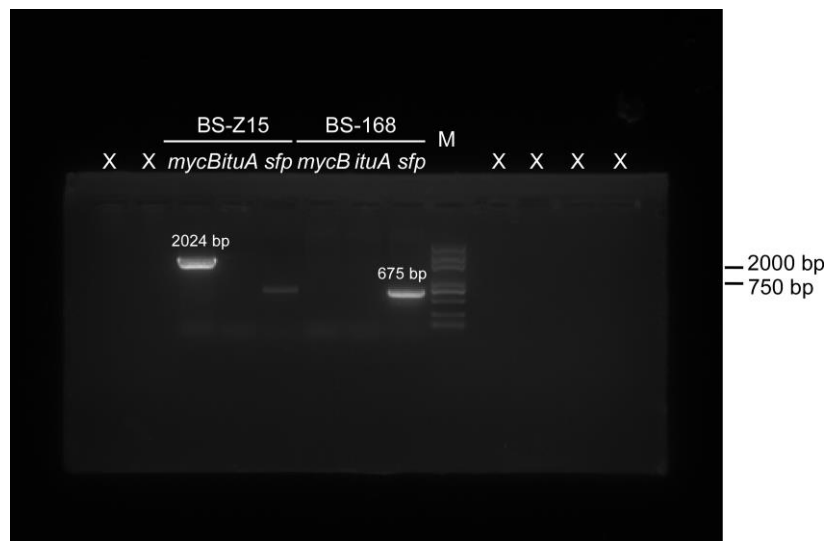

Supplement: S1 File — (PDF) [file pone.0269861.s010.pdf]
