# Supplementary material for: Isolation and characterization of a mycosubtilin homologue antagonizing Verticillium dahliae produced by Bacillus subtilis strain Z15
Source: PLoS One. 2022 Jun 13;17(6):e0269861. doi: 10.1371/journal.pone.0269861 (PMC9191732; doi:10.1371/journal.pone.0269861)
Supplement: S1 Table — (PDF) [file pone.0269861.s008.pdf]

S1 Table. Primers used for genomic PCR and qRT-PCR.

Type	Primer Name	Sequences (5'-3')
Genomic PCR	*MycB-F*	ATGTCGGTGTTTAAAAATCAAGTAACG
	MycB-R	TTAGGACGCCAGCAGTTCTTCTATTGA
	ItuA-F	ATGTATACCAGTCAATTCC
	ItuA-R	GATCCGAAGCTGACAATAG
	Sfp-F	ATGAAGATTTACGGAATTTA
	Sfp-R	TTATAAAAGCTCTTCGTACG
qRT-PCR	*mycA-N-F*	CACCGAGGGTTAGCCAATTA
	mycA-N-R	GACGGACGAGTATAAAGGGAAG
	mycB-Y-F	TACGGTGTTCAAGCCGATAC
	mycB-Y-R	CAGGGTCAAGAGGCACATAA
	mycC-N-F	GGCTCTACAGGAAAGCCTAAAG
	mycC-N-R	CCTGTCAGCCCACCAAATATAA
	16S rDNA-F	AGAGTTTGATCCTGGCTCAG
	16S rDNA-R	CGGTTACCTTGTTACGACTT
	ICS1-F	ATGGATGAATGGGTGCGAAGG
	ICS1-R	AAGAATGCCAGAGGTAAGAGGAGGA
	PR1-F	ACCTCAACGCTCACAACACA
	PR1-R	GGTCCACTGGAGTGCACAAG
	PAL-F	CGGCCATTTCTCGTCGAAAC
	PAL-R	ATAAGTGTCTGTGCCACGGG
	ODD-F	AGCTGCTATGAAGCTACCCG
	ODD-R	AAGCACCCGTATTGCTCGAA
	18S rDNA-F	CCATAAACGATGCCGACCAG
	18S rDNA-R	AGCCTTGCACCATACTCCC

F, forward primer; R, reverse primer.
