# Supplementary material for: Isolation and characterization of a mycosubtilin homologue antagonizing Verticillium dahliae produced by Bacillus subtilis strain Z15
Source: PLoS One. 2022 Jun 13;17(6):e0269861. doi: 10.1371/journal.pone.0269861 (PMC9191732; doi:10.1371/journal.pone.0269861)
Supplement: S2 Table — The four ORFs of Mycosubtilin synthase genes from BS-Z15 were translated to FenF, MycA, MycB and MycC proteins, then analysed by NRPSsp based on the adenylation domain. The start position, end position and predicted substrate were showed in the table. FenF protein has no any predicted adenylation domain. (PDF) [file pone.0269861.s009.pdf]

S2 Table. Predicated amino acid based on NRPS substrate predictor (NRPSsp) in BS-Z15 genome. The four ORFs of Mycosubtilin synthase genes from BS-Z15 were translated to FenF, MycA, MycB and MycC proteins, then analysed by NRPSsp based on the adenylation domain. The start position, end position and predicted substrate were showed in the table. FenF protein has no any predicted adenylation domain.

Protein Identified	Adenylation domain start position	Adenylation domain end position	Substrate
**MYCA** (3971)	2966	3364	N
**MYCB** (5365)	289	686	Y
**MYCB** (5365)	1799	2197	N
**MYCB** (5365)	3310	3718	Q
**MYCB** (5365)	4359	4758	P
**MYCC** (2611)	289	697	S
**MYCC** (2611)	1809	2207	N
